# The prevalence of metabolic syndrome and its predominant components among pre-and postmenopausal Ghanaian women

**DOI:** 10.1186/1756-0500-6-446

**Published:** 2013-11-08

**Authors:** Fareed Kow Nanse Arthur, Michael Adu-Frimpong, James Osei-Yeboah, Faustina Obu Mensah, Lawrence Owusu

**Affiliations:** 1 Department of Biochemistry and Biotechnology, College of Science, Kwame Nkrumah University of Science and Technology, Kumasi, Ghana; 2 Department of Medical Laboratory Technology, College of Health, Kintampo, Ghana; 3 Department of Medical Biochemistry and Molecular Biology, Dalian Medical University, 9 Western Section, Lvshun South Street Lvshunkou District 116044, Dalian City, PR China

**Keywords:** MetS, Postmenopausal, Adiposity, Premenopausal, Obesity, Cardiovascular disease

## Abstract

**Background:**

Metabolic Syndrome (MetS) is a clump of risk factors for development of type 2 diabetes mellitus and cardiovascular diseases. Menopause and age are thought to predispose women to the development of metabolic syndrome. This study aimed to estimate the prevalence of MetS and identify its predominant components among pre-and postmenopausal women in the Kumasi Metropolis, Ghana.

Two hundred and fifty (250) Ghanaian women were randomly selected for the study. They were evaluated for the prevalence of metabolic syndrome using the World Health Organization (WHO), National Cholesterol Education Program Adult Treatment Panel III (NCEP ATP III), International Diabetes Federation (IDF) and Harmonization (H_MS) criteria.

**Results:**

Out of the total subjects, 143 (57.2%) were premenopausal and 107 (42.8%) menopausal. The study population was between the ages of 20–78 years. The overall percentage prevalence of MetS were 14.4%, 25.6%, 29.2% and 30.4% according to the WHO, NCEP-ATP III, IDF and H_MS criteria, respectively. The prevalence was found to increase with age, irrespective of criterion used. Generally, MetS was significantly higher among postmenopausal women (*p* < 0.05 by all criteria) compared to their premenopausal cohort, but with marked inter-criteria variations. Abdominal obesity, blood pressure, fasting blood glucose, triglyceride, very low density lipoprotein cholesterol, and triglyceride-high density lipoprotein cholesterol ratio were significantly (*p* < 0.05) different among the two groups of women.

Central obesity, higher blood pressure and raised fasting blood glucose were the predominant components that contributed to the syndrome in Ghanaian women.

**Conclusion:**

The higher prevalence of the metabolic syndrome in postmenopausal women is an indication that they are at risk of developing cardiovascular disease and type 2 diabetes. Therefore women in that group should be monitored for the two conditions and also be advised to adopt healthy lifestyles to minimize the incidence of these conditions.

## Background

Chronic health problems such as cardiovascular diseases and type 2 diabetes have become major public health concerns worldwide including Ghana [1].

Metabolic syndrome, also known as Syndrome X, Insulin Resistance Syndrome or Dysmetabolic Syndrome, is a common condition that predisposes individuals to the risk of developing cardiovascular diseases and type 2 diabetes. The syndrome is the assemblage of risk factors such as central obesity, high blood pressure, hyperglycaemia, impaired glucose tolerance, hypertriglyceridaemia as well as low levels of high density lipoprotein cholesterol [2]. It is estimated that about 20–25 per cent of the world’s population have the metabolic syndrome and are three times more likely to die from heart attack or stroke compared with people without the syndrome [3]. The risk of cardiovascular diseases assigned to metabolic syndrome seems to be particularly high in women with the estimation that half of all cardiovascular events in women are linked to metabolic syndrome [4].

The aetiology of the syndrome is not clearly defined, but it is linked to visceral obesity [5]. Hence, the theory of metabolic changes in postmenopause and increased abdominal obesity as a result of decrease in oestrogen production is one of the hypotheses that is used to explain the increased incidence of the syndrome during this period [6].

The prevalence of the syndrome differs greatly in different populations. Amongst pre-and postmenopausal women it ranges from 13.8% to more than 60.0% [7-11]. Neto *et al*., [10] estimated prevalence of the syndrome among Brazilian pre-and postmenopausal women to be 24.0% and 44.4% according to NCEP ATP III criterion. Similarly, the prevalence of the syndrome was 13.8% and 54.6% among Korean pre-and postmenopausal women respectively [7]. It is postulated to increase with age with about 40-50% occurring in postmenopausal women [12]. This increase has also been ascribed to menopause and ethnicity [10,13]. Lifestyle modifications such as increase in physical activity and consumption of low caloric diets coupled with reduced intake of alcoholic beverages have been shown to alter the prevalence and gravity of the syndrome as well as to downgrade insulin resistance in women [14,15].

The Ghana Demographic and Health Surveys (GDHS) indicated that the percentage of women aged 15–49 years being overweight or obese grew from 25% to 30% between 2003 and 2008 with the highest values among urban women [16]. There is paucity of data on the prevalence of the syndrome among pre-and postmenopausal women in developing countries, such as Ghana. However, the increasing adaptation of “western” lifestyle coupled with the increased tendency of weight gain after birth in Ghanaian women warrant such a study. The principal components of this syndrome also need to be locally documented as a result of differences in the cut-off levels of the predominant components of metabolic syndrome in Ghanaian women and that of other countries [17]. The significance of these inconsistencies is the yield of various criteria which could affect diagnosis of metabolic syndrome in the same population. There is no data on the prevalence of metabolic syndrome and its predominant components among pre-and postmenopausal women in Ghana. Also no study in Ghana had compared the output of four diagnostic criteria for defining metabolic syndrome in the above population. There is the need to assess the agreement of these criteria in Ghanaian women.

This study, therefore, sought to determine the prevalence of metabolic syndrome and its predominant components among pre-and postmenopausal Ghanaian women in the Kumasi Metropolis, Ashanti Region, Ghana using WHO, NCEP-ATP III, IDF and H_MS diagnostic criteria.

## Methods

### Subjects

This cross-sectional study was carried out between May to July, 2011 in the outpatient department of Suntreso and Seventh Day Adventist Government Hospitals in Kumasi, Ghana. Two hundred and fifty patients were randomly recruited, of which one hundred and forty-three (143) were premenopausal women (control) and one hundred and seven (107) postmenopausal. Women who were still menstruating irrespective of the regularities of their menses were considered as premenopausal women while postmenopausal women were women who had ceased menstruation for at least one year [18]. The participation of the women was voluntary. Informed consent was obtained from each of them after thorough explanation of the study was done in a language they understand. All biochemical analyses were performed without knowledge of subject’s clinical status by means of code numbering. The study was approved (CHRPE/ KNUST/ KATH/ 01_02_11) by the local Committee on Human Research Publication and Ethics.

### Sample size consideration

The 250 participants were used after the estimation of minimum sample sizes for both pre-(98) and postmenopausal women (88) to achieve 80% power based on the method as suggested by [19,20]. The parameters used were prevalence of metabolic syndrome among pre-and postmenopausal women [10]; 24% and 44.4% respectively, confidence interval of 95%, relative sample size of 0.9, probability of type II error, 20% and probability of type I error 5%.

### Exclusion criteria

Since metabolic syndrome is a constellation of interrelated risk factors including hypertension, dyslipidaemia [(low levels of HDL-C, elevated triglycerides (TG)], obesity, insulin resistance and elevated blood glucose, women with diseases/conditions that could affect the diagnosis of metabolic syndrome were excluded. The health history of each of the women was obtained from their hospital folders and those found to be pregnant, diabetics, hypertensive as well as having polycystic ovarian syndrome, fatty liver and cancer were not recruited into the study.

### Questionnaire

Self-reported questionnaire were administered to determine menopause status, smoking status, alcohol intake, educational level, physical activity levels, occupation and family medical history. Passive smokers were women who were exposed to cigarette smokes by their relatives and/or husbands. Occupation was categorized into manual (traders, farmers and seamstress), non-manual (civil servants) and out of economically active population (unemployed). Physically inactive women were those who did not exercise at all within a week whilst alcoholics were defined as the participants that consumed alcohol on a daily basis.

### Measurement of anthropometric variables

Anthropometric measurements included height to the nearest centimetre without shoes and weight to the nearest 0.1 kg in light clothing. Subjects were weighed on a bathroom scale (BR9012, Zhongshan Camry Electronic Co. Ltd, Guangdong, China) and their height measured with a wall-mounted ruler. Body mass index (BMI) was calculated by dividing weight (kg) by height squared (m2). Waist circumference was measured at the midpoint between the last rib and the iliac crest with the participants standing and wearing light cloths with a Gulick II spring-loaded measuring tape (Gay Mills, WI). The hip circumference was measured at the widest level over the greater trochanters [21] and the waist-to-hip ratio (WHR) calculated by dividing the waist circumference (cm) by the hip circumference (cm). Thigh circumference (THC) on the other hand was measured on the left leg below the gluteal fold and waist-to-thigh ratio (WTR) calculated by dividing waist circumference (cm) by the thigh circumference (cm). The WHR, WHtR and WTR were recorded to the nearest 2 decimal places. All measurements were taken thrice and the means were recorded.

### Blood pressure (using Korotkoff 1 and 5)

Blood pressure was taken by trained personnel with participants in sitting position and having rested for at least 10 minutes using sphygmomanometer with appropriate cuff sizes and stethoscope in accordance with the recommendation of the American Heart Association [22]. Triplicate readings were taken per subject, after two minutes intervals and the mean value was recorded to the nearest 2.0 mm H. Systolic blood pressure (SBP) and diastolic blood pressure (DBP) were taken at the 1st and 5th Korotkoff sounds respectively. Pulse pressure (PP) was calculated using SBP-DBP.

### Definition of metabolic syndrome

The definitions for the four diagnostic criteria applied in this study are shown in Table 1.

**Table 1:** Definition of Metabolic Syndrome

				**Risk factors**					
**MetS definition/criteria**	**WC/CO (cm)**	**WHR**	**BMI (kg/m**^**2**^**)**	**BP (mmHg)**	**TG (mmol/ L)**	**HDL-C (mmol/ L)**	**FBS (mmol/ L)**	**Remark(s)**	**Ref**
*NCEP-ATP III*	> 88			≥ 130/85	≥ 1.7	< 1.29	≥ 5.6	Any 3 or more factors	[22]
*IDF*	> 80*			≥ 130/85	≥ 1.7	< 1.29	≥ 5.6	WC and any other 2 factors	[3]
*H_MS*	> 80			≥ 130/85	≥ 1.8	< 1.30	≥ 5.7	Any 3 or more factors	[23,24]
*WHO*		> 0.85	> 30	≥ 140/90	≥ 1.7	< 1.01	≥ 6*.*1*	Impaired glucose tolerance/insulin resistance/DM II and any other 2 factors	[25]

*: a principal component of definition, DM: diabetes mellitus, WHO = World Health Organization; ATP III = Adult Treatment Panel III; IDF = International Diabetes Federation; H_MS: Harmonization, IGT = impaired, glucose tolerance; impaired fasting glycaemia; T2DM = Type 2 diabetes mellitus; BMI = body mass index; WC = waist circumference; TG = triglycerides; HDL-C = high-density lipoprotein cholesterol; WC/CO: waist circumference/central obesity, WHR: waist-to-hip ratio, BMI: Body Mass Index, BP: Blood Pressure, TG: Triglycerides, HDL-C: High Density Lipoprotein Cholesterol, FBS: Fasting Blood Sugar.

Four categories of BMI (≤20, 20–24.9, 25–29.9, and ≥30 kg/m2) were also identified. The categories were selected according to WHO recommendations to define individuals with a healthy weight (BMI 20–24.9), overweight (BMI 25–29.9) and obese (BMI ≥ 30). Individuals with a BMI ≤20 kg/m^2^ were classified as underweight. Women with WHR < 0.80, 0.80-0.84 and ≥ 0.85 were classified as normal weight, overweight or obese respectively; women with WHtR = < 0.53 and > 0.53 were classified as normal and obese respectively; Hyperglycaemia = fasting blood sugar ≥ 6.1 mmol/l, Impaired Fasting Glucose = fasting blood sugar between 6.1 to 6.9 mmol/l and Dyslipidaemia = TG >150 mg/dl and HDL-C < 40 mg/dl.

### Blood sampling, processing and analyses

#### Biochemical analyses

Venous blood samples were collected after overnight fast (12–16 hours) between 7 am and 10 am. About 5 ml of venous blood was collected; 4 ml was dispensed into vacutainer® plain tubes and 1 ml into fluoride oxalate tubes. After centrifugation at 500 g for 15 minutes, the serum and plasma were stored at − 80°C until assayed. Parameters that were determined included: fasting blood glucose (FBG), total cholesterol (TC), triglycerides (TG) and high density lipoprotein cholesterol (HDL-C) according to reagents manufacturer’s specification (Fortress Diagnostics Limited, Unit 2C Antrim Technology Park, Antrim BT41 1QS, UK). Serum low density lipoprotein cholesterol (LDL-C) and very low density lipoprotein (VLDL-C) were calculated using the Frederickson-Friedwald’s formula [23].

### Statistical analysis

Normality of all variables was tested and found to be normal before the statistical analyses using the D’ Agostino-Pearson procedure. Continuous variables are expressed as their mean ± SEM, while categorical variables are expressed as proportion. Comparisons of the general characteristics of postmenopausal women against the premenopausal group were performed using unpaired *t* tests, χ ^2^ tests, or Fisher exact tests where appropriate. A *p* < 0.05 was considered statistically significant for comparisons. The agreement between WHO, NCEP ATP III, IDF and H_MS criteria of metabolic syndrome was determined by the kappa statistics (k). The level of agreement is considered poor when k ≤ 0.20, fair k = 0.21 to 0.40, moderate k = 0.41 to 0.60, good k = 0.61 to 0.80, and very good k = 0.80 to 1.00 [24]. P values of less than 0.05 were considered statistically significant. GraphPad Prism version 5.00 and MedCalc version 12.3.2 for Windows were used for statistical analysis (GraphPad software, San Diego California USA, http://www.graphpad.com; MedCalc software bvba, MedCalc Software, Acacialaan 22, B-8400 Ostend, Belgium, http://www.medcalc.org).

## Results

### General characteristics of the population

Out of 250 women, 143 (57.2%) were premenopausal and 107 (42.8%) were postmenopausal. The general characteristics of the study population are shown in Table 2. The mean age of postmenopausal (57.25 ± 0.8) was significantly higher (*p* < 0.0001) than the mean age of the premenopausal (34.48 ± 0.7) women. Postmenopausal women had significantly (*p* < 0.0001) larger waist circumference, higher mean WHR, WTR, WHtR, SBP, DBP and PP than their premenopausal counterpart. The two groups did not however differ significantly (*p* = 0.415) in BMI values as indicated in Table 2. Moreover, the proportion of postmenopausal women who were passive smokers and/or drank alcoholic beverage was significantly more (*p* = 0.016 and 0.023, respectively). A significantly higher (*p* = 001) proportion of the postmenopausal women were unemployed whereas premenopausal cohorts were mainly (*p* = 0.0006) non-manual workers (Table 2). On the other hand, the proportion of postmenopausal subjects with family history of diabetes was more than premenopausal women (*p* = 0.042).

**Table 2:** General characteristics of Ghanaian pre-and postmenopausal women

**Parameters**	**Total**	**Postmenopausal**	**Premenopausal**	***P value***
	**(n = 250)**	**(n = 107)**	**(n = 143)**	
Age (years)	44.23 ± 0.90	57.25 ± 0.80	34.48 ± 0.74	<0.0001
***Anthropometric Parameters***				
WC (cm)	92.41 ± 0.72	95.83 ± 0.93	89.85 ± 1.01	<0.0001
WHR	0.88 ± 0.004	0.91 ± 0.005	0.87 ± 0.005	<0.0001
WTR	1.67 ± 0.009	1.72 ± 0.013	1.62 ± 0.011	<0.0001
WHtR	0.58 ± 0.004	0.60 ± 0.006	0.56 ± 0.006	<0.0001
BMI (kg/m^2^)	26.65 ± 0.32	26.92 ± 0.46	26.39 ± 0.44	0.4150
***Hemodynamic Parameters***				
SBP (mmHg)	132.5 ± 1.20	140.6 ± 1.75	126.4 ± 1.44	<0.0001
DBP (mmHg)	86.3 ± 0.73	89.87 ± 1.09	83.62 ± 0.93	<0.0001
PP (mmHg)	46.17 ± 0.76	50.76 ± 1.12	42.73 ± 0.93	<0.0001
***Socio-demographic Parameters***				
Passive Smokers	35(14%)	22(20.6%)	13(9.1%)	0.0160
Alcoholics	89(35.6%)	47(43.9%)	42(29.4%)	0.0230
Married	148(59.2%)	61(57.01%)	87(60.8%)	0.6033
Occupation				
Manual	112(44.8%)	52(48.6%)	58(40.6%)	0.2470
Non-manual	118(47.2%)	39(36.4%)	81(56.6%)	0.0020
Outside of EAP	20(8%)	16(15%)	4(2.8%)	0.0006
High Education	73(29.2%)	27(25.2%)	46(32.2%)	0.2621
Physically Inactive	102(40.8%)	37(36.4%)	65(45.5%)	0.0920
Family History of Hypertension	95(38%)	41(38.3%)	54(37.8%)	1.0000
Family History of Diabetes	65(26%)	35(32.7%)	30(21%)	0.0420
***Biochemical Assays***				
TC (mmol/l)	4.40 ± 0.053	4.41 ± 0.084	4.40 ± 0.069	0.8504
TG (mmol/l)	1.20 ± 0.033	1.31 ± 0.061	1.12 ± 0.035	0.0060
HDL-C (mmol/l)	1.34 ± 0.016	1.31 ± 0.027	1.37 ± 0.021	0.0700
LDL-C (mmol/l)	2.51 ± 0.048	2.50 ± 0.076	2.52 ± 0.061	0.9131
VLDL-C (mmol/l)	0.42 ± 0.012	0.46 ± 0.022	0.40 ± 0.013	0.0060
HDL-C/TC	0.31 ± 0.004	0.30 ± 0.006	0.32 ± 0.005	0.0702
TG/HDL-C	0.97 ± 0.042	1.11 ± 0.081	0.86 ± 0.039	0.0040
FBG (mmol/l)	5.19 ± 0.079	5.57 ± 0.15	4.9 ± 0.074	<0.0001

Continuous data were presented as mean ± standard error of mean (SEM). Categorical data are presented as frequency with percentages in parenthesis. Continuous data were compared using unpaired t-test whilst categorical data were compared using Fischer’s exact test. VLDL-C: Very Low Density Lipoprotein-Cholesterol, LDL-C: Low Density Lipoprotein-Cholesterol, HDL-C: High Density Lipoprotein, TC: Total Cholesterol, TG: Triglycerides, FBG: Fasting Blood Glucose, WHR: Waist-to-Hip Ratio, BMI: Body Mass Index, WC: Waist Circumference, WTR: Waist-to-Thigh Ratio, WHtR: Waist-to-Height Ratio, SBP: Systolic Blood Pressure, DBP: Diastolic Blood Pressure, PP: Pulse Pressure, EAP: Economically active population.

Biochemical analyses point to a significant (p < 0.05) increase in the mean level of TG, VLDL cholesterol, and fasting blood glucose among postmenopausal participants as compared to the premenopausal subjects, and the mean TG/HDL-C ratio was also higher among the postmenopausal group (Table 2). Though these biochemical markers were significantly raised in postmenopausal women, they were within normal range. The postmenopausal women however had reduced HDL-C, though not statistically significant, compared to their premenopausal counterparts (Table 2).

The percentage prevalence of MetS was 14.4%, 25.6%, 29.2% and 30.4% using WHO, NCEP ATP III, IDF and H_MS criteria respectively for the total population. The prevalence was higher among the postmenopausal group (i.e. 25.2%, 41.1%, 43.0% and 43.9% for WHO, NCEP ATP III, IDF and H_MS respectively) compared to the premenopausal population (i.e. 6.3%, 14.7%, 18.9% and 23.1% respectively for WHO, NCEP ATP III, IDF and H_MS criteria respectively) as shown in Table 3.

**Table 3:** Prevalence of metabolic syndrome and metabolic score among Ghanaian pre-and postmenopausal women

**Parameters**	**Total**	**Postmenopausal**	**Premenopausal**	***P value***
	**(n = 250)**	**(n = 107)**	**(n = 143)**	
**Prevalence of metabolic syndrome**				
WHO	36(14.4%)	27(25.2%)	9(6.3%)	<0.0001
NCEP-ATP III	65(26.0%)	44(41.1%)	21(14.7%)	<0.0001
IDF	73(29.2%)	46(43.0%)	27(18.9%)	<0.0001
H_MS	76(30.4%)	47(43.9%)	29(20.3%)	<0.0001
**Prevalence of clustering of one or two or more components of metabolic syndrome**				
*WHO*				
0	34(13.6%)	1(1.0%)	33(23.1%)	<0.0001
1	70(28.0%)	20(18.7%)	50(35.0%)	0.0050
2	76(30.4%)	45(42.1%)	31(21.7%)	0.0010
>2 without MetS	34(13.6%)	14(13.1%)	20(14.0%)	1.0000
*NCEP-ATP III*				
0	30(12.0%)	3(2.8%)	27(18.9%)	<0.0001
1	57(22.8%)	13(12.1%)	44(30.8%)	0.0010
2	98(39.2)	47(43.9%)	51(35.7%)	0.1930
*IDF*				
0	13(5.2%)	0(0.0%)	13(9.1%)	0.0010
1	50(20.0%)	8(7.5%)	42(29.4%)	<0.0001
2	111(44.4%)	52(48.6%)	59(41.3%)	0.3030
>2 without MetS	3(1.2%)	1(1.0%)	2(1.4%)	1.0000
*H_MS*				
0	13(5.2%)	0(0.0%)	13(9.1%)	0.0010
1	50(20.0%)	8(7.5%)	42(29.4%)	<0.0001
2	111(44.4%)	52(48.6%)	59(41.3%)	0.3030

Data are presented as a proportion with corresponding percentages in parenthesis. The proportions were compared using Fischer’s exact test. Mets: Metabolic Syndrome. WHO: World Health Organization, NCEP-ATP III: National Cholesterol Education Program-Adult Treatment Panel III, IDF: International Diabetes Federation and H_MS: Harmonization.

In terms of proportion, women without any MetS risk factor (i.e. zero metabolic score) were significantly higher (*p* < 0.0001 using WHO and NCEP ATP III criteria, and *p* = 0.001 using the IDF and H_MS criteria respectively) among the premenopausal subjects as compared to the postmenopausal participants using all criteria. A metabolic score of 1 was found to be more associated with the premenopausal group than their postmenopausal counterparts (Table 3). However, the ratio of women who were about to traverse to the MetS zone (i.e. metabolic score of 3) was similar between the postmenopausal group and the premenopausal individuals with the exception of WHO criteria (Table 3). There were some women who possessed three or more MetS risk factors, yet they did not exhibit the syndrome according to WHO and IDF criteria. This small population was common among the premenopausal group, though not significant (Table 3).

The prevalence of abdominal (central) obesity [87.9% in postmenopausal (WHO), 80.4% in postmenopausal (NCEP ATP III); 95.3% in postmenopausal (IDF and H_MS)]; raised fasting blood glucose (36.5% in postmenopausal); and raised blood pressure (83.2% in postmenopausal) were significantly higher (*p* < 0.0001 for all) compared to the premenopausal population [i.e. 56.0% (WHO), 51.7% (NCEP ATP III) and 79.0% (IDF and H_MS); 6.3% (WHO), 16.1% (NCEP, IDF &H_MS); 31.5% (WHO) and 49.7% (NCEP, IDF & H_MS)] for abdominal obesity, raised fasting blood glucose and raised blood pressure respectively as shown in Table 4. These components contributed to higher prevalence of MetS (about tenfold) among the postmenopausal group compared to the premenopausal individuals using these criteria.

**Table 4:** Prevalence of the determinant components of metabolic syndrome among Ghanaian pre-and postmenopausal women

**Parameters**	**Total**	**Postmenopausal**	**Premenopausal**	***P value***
	**(n = 250)**	**(n = 107)**	**(n = 143)**	
***WHO***				
Central Obesity	177(70.8%)	94(87.9%)	83(58.0%)	<0.0001
Raised Fasting Glucose	39(15.6%)	30(28.0%)	9(6.3%)	<0.0001
Raised Triglyceride	24(9.6%)	15(14.0%)	9(6.3%)	0.0507
Raised Blood Pressure	109(43.6%)	64(59.8%)	45(31.5%)	<0.0001
Reduced HDL-C	47(18.8%)	26(24.3%)	21(14.7%)	0.0710
***NCEP ATP III***				
Abdominal Obesity	160(64.0%)	86(80.4%)	74(51.7%)	<0.0001
Raised Fasting Glucose	62(24.8%)	39(36.5%)	23(16.1%)	0.0003
Raised Triglyceride	24(9.6%)	15(14.0%)	9(6.3%)	0.0507
Raised Blood Pressure	160(64.0%)	89(83.2%)	71(49.7%)	<0.0001
Reduced HDL-C	91(36.4%)	45(42.1%)	46(32.2%)	0.1132
***IDF***				
Abdominal Obesity	215(86.0%)	102(95.3%)	113(79.0%)	0.0002
Raised Fasting Glucose	62(24.8%)	39(36.5%)	23(16.1%)	0.0003
Raised Triglyceride	24(9.6%)	15(14.0%)	9(6.3%)	0.0507
Raised Blood Pressure	160(64.0%)	89(83.2%)	71(49.7%)	<0.0001
Reduced HDL-C	91(36.4%)	45(42.1%)	46(32.2%)	0.1132
***H_MS***				
Abdominal Obesity	215(86.0%)	102(95.3%)	113(79.0%)	0.0002
Raised Fasting Glucose	62(24.8%)	39(36.5%)	23(16.1%)	0.0003
Raised Triglyceride	24(9.6%)	15(14.0%)	9(6.3%)	0.0507
Raised Blood Pressure	160(64.0%)	89(83.2%)	71(49.7%)	<0.0001
Reduced HDL-C	91(36.4%)	45(42.1%)	46(32.2%)	0.1132

Data are presented as proportion with corresponding percentages in parenthesis. The proportions were compared using Fischer’s exact test.

On the other hand, using the NCEP criterion, the contributing factors were raised blood pressure, central obesity and raised fasting blood glucose (Table 4). The percentage prevalence of these components was raised blood pressure (83.2%), central obesity (80.4%) and raised fasting blood glucose (36.5%) in postmenopausal women and were statistically significant (p < 0.05) compared to premenopausal subjects (Table 4).

The prevalence of BMI overweight, WHR obesity, WHtR obesity, hyperglycaemia, diabetes and hypertension were significantly (*p* < 0.05) higher among postmenopausal group whilst WHR overweight, WHR obesity and WHtR normal were significantly (*p* < 0.0001) higher among premenopausal subjects than their postmenopausal counterparts (Table 5). BMI obesity was prevalent in premenopausal women than their postmenopausal cohorts though not significant (Table 5).

**Table 5:** Prevalence of obesity, hypertension, diabetes and dyslipidaemia among population classified by menopause

**Parameters**	**Total**	**Postmenopausal**	**Premenopausal**	***P value***
	**(n = 250)**	**(n = 107)**	**(n = 143)**	
***BMI***				
Underweight	23(9.2%)	07(6.5%)	16(11.2%)	0.2700
Normal	80(32.0%)	32(29.9%)	48(33.6%)	0.5850
Overweight	89(35.6%)	46(43.0%)	43(30.1%)	0.0450
Obese	58(23.2%)	22(20.6%)	36(25.2%)	0.4500
***WHR***				
Normal	16(6.4%)	01(1.0%)	15(10.5%)	0.0020
Overweight	42(16.8%)	06(5.6%)	36(25.2%)	<0.0001
Obese	192(76.8%)	100(93.5%)	92(64.3%)	<0.0001
***WHtR***				
Normal	66(26.4%)	18(16.8%)	48(33.6%)	0.0040
Obese	184(73.6%)	89(83.2%)	95(66.4%)	0.0040
**FBG**				
Hyperglycaemia	40(16.0%)	30(28.0%)	10(7.0%)	<0.0001
Impaired Glucose	12(4.8%)	06(5.6%)	06(4.2%)	0.7670
**DYSLIPIDAEMIA**	16(6.4%)	09(8.4%)	07(4.9%)	0.3020

Data are presented as proportion with corresponding percentages in parenthesis. The proportions were compared using Fischer’s exact test. n: number of subjects, Four categories of BMI (≤20, 20–24.9, 25–29.9, and ≥30 kg/m2) were identified. The categories were selected according to WHO recommendations to define individuals with a healthy weight (BMI 20–25), overweight (BMI 25–29.9) and obese (BMI ≥ 30). Individuals with a BMI ≤20 kg/m2 were classified as underweight. Women with WHR < 0.80, 0.80-0.84 and ≥ 0.85 were classified as normal weight, overweight or obese respectively, women with WHtR = < 0.53 and > 0.53 were classified as normal and obese respectively; Hyperglycaemia = fasting blood sugar ≥ 6.1 mmol/l, Impaired Glucose = fasting blood sugar between 6.1 to 6.9 mmol/l and Dyslipidaemia = TG >150 mg/dl and HDL-C < 40 mg/dl.

The degree of agreement (kappa statistic) between the four diagnostic criteria is depicted in Table 6. There kappa statistic between H_MS total/IDF total, H_MS post/IDF post, H_MS pre/IDF pre, H_MS pre/NCEP pre and IDF pre/NCEP pre were 0.97, 0.98, 0.96, 0.76 and 0.70 respectively. The overall degree of agreement between WHO/NCEP, WHO/IDF and WHO/H_MS were 0.53, 0.47 and 0.47 respectively.

**Table 6:** Degree of agreement between WHO, NCEP ATP III, IDF and H_MS definitions in diagnosing metabolic syndrome (Kappa Statistic)

**Criteria**	**NCEP total**	**NCEP post**	**NCEP pre**	**IDF total**	**IDF post**	**IDF pre**	**H_MS total**	**H_MS post**	**H_MS pre**
**WHO total**	0.53 ± 0.08			0.47 ± 0.06			0.47 ± 0.06		
**WHO post**		0.53 ± 0.08			0.46 ± 0.08			0.44 ± 0.08	
**WHO pre**			0.56 ± 0.11			0.39 ± 0.10			0.42 ± 0.10
**IDF total**	0.54 ± 0.08								
**IDF post**		0.54 ± 0.08							
**IDF pre**			**0.70 ± 0.08**						
**H_MS total**	0.56 ± 0.08			**0.97 ± 0.02**					
**H_MS post**		0.56 ± 0.08			**0.98 ± 0.02**				
**H_MS pre**			**0.76 ± 0.07**			**0.96 ± 0.03**			

Data are presented as weighted Kappa ± standard error (SE). WHO total, WHO post and WHO pre: definition of metabolic syndrome in the total population, postmenopausal and premenopausal group respectively using WHO criterion, NCEP total, NCEP post and NCEP pre: definition of metabolic syndrome in the total population, postmenopausal and premenopausal group respectively using NCEP criterion, IDF total, IDF post and IDF pre: definition of metabolic syndrome in the total population, postmenopausal and premenopausal group respectively using IDF criterion, H_MS total, H_MS post and H_MS pre: definition of metabolic syndrome in the total population, postmenopausal and premenopausal group respectively using H_MS criterion.

**Figure 1:**
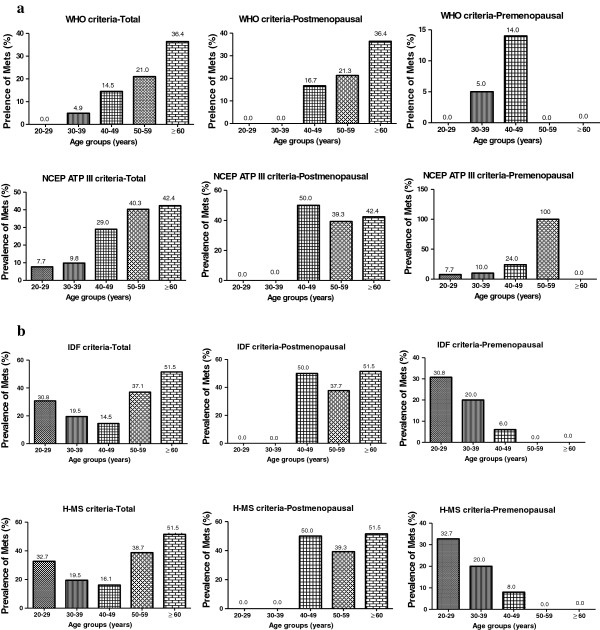
**a: Prevalence of MetS Stratified by Menopausal Status and Age (WHO and NCEP-ATP III Criteria). b**: **Prevalence of MetS Stratified by Menopausal Status and Age (IDF and H_MS Criteria).**

The prevalence of MetS generally increased with age, using chi-square for trend, irrespective of the criteria applied, (Figure 1a and b). Using WHO criterion as an example, the prevalence significantly (χ^2^ = 25.75; *p* <0.0001) increased from 0.0% (0/52) among 20–29 years group to 4.9% (2/41) among 30–39 years group and through to 36.4% (12/33) in ≥ 60 years group (Figure 1a). The age specific distribution of MetS was usually higher among the postmenopausal group. The highest prevalence of MetS among the postmenopausal women was seen in 60 years group or older irrespective of the criteria with the exception of NCEP ATP III which was seen among 40–49 years group (Figure 1a).

## Discussion

MetS has become a public health problem due to its link to diseases such as ischemic heart disease, stroke, dementia, non-alcoholic steatohepatitis, polycystic ovarian disease, haemochromatosis, endometrial and oesophageal cancers [25]. Even though the concept was coined several decades ago, the comparison of prevalence between populations was made possible after the standardization of diagnostic criteria. The differences in genetic profile, lifestyle factors such as eating habits and level of physical activity, age, menopausal status and gender determine the prevalence and the predominant components of MetS in a population [26].

The present study assessed the prevalence of MetS and its predominant components among pre- and postmenopausal Ghanaian women. The prevalence of MetS as observed in this study increased from 14.4% through 25.6% to 29.2% and further to 30.4% when the WHO, NCEP ATP III, IDF and H_MS criteria respectively were used (Table 3). The prevalence of MetS varies among women and depends on the characteristics of the population as well as the diagnostic criteria applied [18,27]. This study considered four different diagnostic criteria and each gave different degree of prevalence and these results are in agreement with studies carried out among Brazilian, Chinese, German and Korean women which estimated the prevalence of MetS, to range from 10.7% through 20.9% and 33.7% to 36.1% [2,10,18,27-29]. The degree of agreement between H_MS and IDF criteria was very good, (weighted kappa = 0.97). The overall degree of agreement between WHO/NCEP, WHO/IDF and WHO/H_MS was moderate (0.53, 0.47 and 0.47 respectively). Choi and colleagues estimated similar degree of agreement between WHO and NCEP criteria among Korean population [30]; however there was paucity of data comparing the agreement between all the four criteria together.

When WHO diagnostic criterion was used, the prevalence of MetS among postmenopausal women was higher (25.2%) compared to their premenopausal counterparts (6.3%) (Table 3). This finding is consistent with a study conducted in 2008 with 200 climacteric (menopausal) women in Pakistan, which found MetS in 21.0% of postmenopausal women against 7.0% of premenopausal women using the same criterion [31]. Piche and colleagues [32] found prevalence of 29.6% among Canadian postmenopausal women using the WHO criterion. Applying the NCEP ATP III criterion, the prevalence of MetS was estimated to be higher among postmenopausal subjects in the present study (41.1%) than their premenopausal counterparts (14.7%). This confirms that the prevalence of MetS can differ in a population depending on the criterion used. This observation is similar to studies carried out among premenopausal Korean and postmenopausal Ecuadorian women respectively which estimated the prevalence of MetS to range from 13.8% to 41.5% using the NCEP ATP III criterion [33,34]. Pandey and colleagues [35] found prevalence of MetS among Indian women to be 56% using NCEP ATP III criterion whereas 33.7% prevalence rate of MetS was observed by Ruan *et al.,*[29] using IDF criterion among Chinese women. Certainly, lifestyle and genetic characteristics of Ghanaian women are likely to be significantly different from women from China and India and these may explain the differences in prevalence rates of MetS obtained.

In addition, the prevalence of MetS was higher among postmenopausal women [(43.0% (IDF) and 43.9% (H_MS)] than premenopausal women [(18.9% (IDF) and 20.3% (H_MS)] (Table 3). Pandey *et al*., [36] used both criteria to estimate higher prevalence of MetS among postmenopausal Indian women compared to their premenopausal counterparts. In their study, the prevalence of MetS among postmenopausal women was higher than in the premenopausal group by both, IDF (premenopausal 45% and postmenopausal 55%) and H_MS criteria (premenopausal 44% and postmenopausal 56%). The differences in the prevalence rates in both present and Indian studies may be explained by differences in socio-cultural practices, lifestyle, as well as genetic compositions. The prevalence of metabolic syndrome in the study population might increase in future as most of the women especially postmenopausal group, had metabolic scores of 2 when all the diagnostic criteria were considered. The findings from this study suggest that the prevalence of MetS is dependent on age regardless of the criteria used. The influence of age on MetS among pre-and postmenopausal women is important and this trend has been established in similar populations elsewhere [10,36]. This explains why postmenopausal women had higher age-specific prevalence of MetS than their premenopausal counterparts when all the four diagnostic criteria were applied (Figure 1a and b). The age-specific prevalence of MetS peaks at ≥60 years for postmenopausal women. These findings are in partial accordance with those observed in Seychellois [37] women where the prevalence of MetS was highest among 55–64 years old. Physical activity and lean muscle mass naturally diminishes with age in women [38]. The body composition of women shifts to more fat and less muscle which slows down the rate at which the body metabolises biomolecules and results in weight gain especially central fatness culminating in metabolic abnormalities and higher MetS prevalence.

Premenopausal Ghanaian women develop MetS earlier (20–29 years) when NCEP ATP III, IDF and H_MS criteria were applied. This result is concurrent with the study conducted by Kim *et al*., [7] which identified the onset of MetS to be 20–29 years among Korean pre-and postmenopausal women. The reason for marked increase in the prevalence of MetS among premenopausal individuals at the age group of 20–29 years (32.7%) and 30–39 (20.0%) is not known, though it is possible these women might have kept positive caloric balance for some time and this had resulted in an increase in their obesity indices, blood pressure and lipid profile. Insulin sensitivity and glucose intolerance are not entirely explained by a woman’s hormonal status. There are now data showing that weight gain in women is a stronger predictor of impaired glucose tolerance than menopausal status [39].

Examination of the Ghanaian women with MetS using the three diagnostic criteria identified central obesity, raised blood pressure and raised fasting glucose (WHO, IDF & H_MS) as the predominant components (Table 4). The components of MetS were common in the postmenopausal group when they were analyzed in relation to menopausal status. Oliveira *et al*., [40] found similar order of frequency of components respectively with the following proportions: central obesity (84.1%), raised blood pressure (53.6%), raised triglycerides (18.1%) and raised fasting glucose (16.7%). However, Oh *et al*., [18] in their study of 449 South Korean women listed the following predominant components: reduced HDL-C, raised blood pressure, raised triglycerides, raised fasting blood glucose and abdominal obesity. Contrarily, NCEP-ATP III criterion identified raised blood pressure, central obesity and raised fasting blood glucose as the predominant components in Ghanaian women. In premenopausal women, fat accumulates in lower extremities, to a greater extent, as a result of oestrogen secretion. After meals, the flow of blood containing high levels of chylomicrons to fat stored in the thighs and hips increases in women, but not in men [41]. Furthermore, the fats stored around the hips and thighs serve as storage form of energy during pregnancy as well as cushion for the reproductive organs [41]. These could be the reasons why women store more fat in their lower body. However, during menopause the pattern of hormone secretion changes and gradually causes fat accumulation in visceral tissues of abdomen which results in central obesity [42]. A lot of metabolic changes in postmenopausal women are related to the decrease in oestrogen secretion and consequent accumulation of abdominal fat. Moreover, central obesity is linked to a greater amount of visceral fat than to lower-body obesity, which is associated with more subcutaneous fat. Visceral fat produces free fatty acids and inflammatory cytokines which directly drains into the portal vein, and is thus likely to have a direct signalling and metabolic relation with the liver in comparison to subcutaneous fat [43,44]. Fat deposits in the liver are associated with the overproduction of very low-density lipoprotein predisposing women to atherogenic dyslipidaemia (elevated triglyceride, low HDL-cholesterol level, and small dense LDL cholesterol particles) [44,45]. Elevated levels of small dense-LDL-cholesterol get entrapped in the endothelium of the arterial wall and are oxidized leading to arterial stiffness and atherosclerosis [46] and these can culminate in high blood pressure and related conditions. Ghanaian women showed abdominal obesity and raised blood pressure especially among postmenopausal group and this may be due to the fact that they generally have adopted western lifestyle of consuming high-energy food whilst undertaking limited physical exercise.

Plasma TG and HDL-cholesterol are known to be inversely correlated from epidemiological studies [47,48]. The enzyme cholesteryl ester transfer protein (CETP) balances the levels of TG and HDL-cholesterol by mediating the transfer of triglycerides (TGs) from TG-rich lipoproteins to HDL and LDL particles in exchange for cholesteryl. esters which leads to low HDL-C and high small dense-LDL-C [49]. It has been proposed that high CETP activity explains some of the high TG levels and low HDL-C levels (dyslipidaemia), observed in persons with MetS [50]. In this study, menopause was associated with an increase in serum triglyceride but mean levels of HDL-cholesterol were similar between premenopausal and postmenopausal women (Table 2), which is consistent with the observation among Korean women by Kim *et al*., [33].

The present study also demonstrated that the prevalence of BMI overweight, WHR obesity, WHtR obesity, hyperglycaemia and hypertension were significantly higher among postmenopausal group compared to the premenopausal population whilst WHR overweight was the reverse. This finding is in disagreement with the study of Jaber *et al*., [51] which listed only low HDL cholesterol and raised fasting glucose as the predominant components of MetS among Arab American women. This could be attributed to differences in genetics and environment. Other studies have shown that the above components are predominant indicators of MetS [2,7,29] however, menopause has been established to increase the risk of women to above-mentioned factors [52,53]. This further buttresses the point that postmenopausal status is an independent risk factor for MetS and all of its individual components [54]. Moreover, postmenopausal women are thought to accumulate more fat in the intra-abdominal depot than do premenopausal women and therefore have a greater risk of developing metabolic complications associated with obesity [55].

The strength of this study is that all variables were measured using standard methods and vigorous quality control. However, our cross sectional study design and small sample size does not allow us to generalize the findings to all women in Kumasi metropolis. Another limitation of our study is the age difference between pre-and postmenopausal women which could also influence the prevalence of metabolic syndrome in both groups. Therefore future prospective studies should be used to confirm the difference in prevalence of metabolic syndrome in large population of age-matched pre-and postmenopausal women in Ghana.

## Conclusion

The findings in this study revealed the presence of metabolic syndrome among Ghanaian women. The syndrome was higher among postmenopausal women, irrespective of diagnostic criteria used. Age had a major influence on the prevalence and the individual constituents of the syndrome. The predominant components identified were central obesity, raised blood pressure and raised fasting blood glucose in the order of frequency. Conscious control of these individual constituents is achievable and must be encouraged in the population. Metabolic syndrome has become a significant health problem in our contemporary world and therefore all efforts should be made to create local awareness, early diagnosis and prevention. This can decrease the burden on our limited health resources, prevent complications such as type 2 diabetes, cardiovascular diseases, and reduce morbidity and early mortality among women. Women, still in their childbearing age, should also be advised to balance their energy intake and expenditure in order to reduce the alarming rate of the syndrome incidence. Moreover, exercise and consumption of low caloric foods will improve plasma lipid concentrations by raising their HDL cholesterol concentrations [56], decrease triglycerides concentrations [57] or both [58]. Furthermore, physical activity is linked with lowered blood pressure, improved glucose intolerance, insulin sensitivity and lowered risk of type 2 diabetes [59].

## Abbreviations

MetS: Metabolic syndrome; H_MS: Harmonization; BMI: Body mass index; WHR: Waist-to-hip ratio; WC: Waist circumference; WTR: Waist-to-thigh ratio; WHtR: Waist-to-height ratio; SBP: Systolic blood pressure; DBP: Diastolic blood pressure; TC: Total cholesterol; TG: Triglyceride; HDL-C: High density lipoprotein cholesterol; LDL-C: Low density lipoprotein cholesterol; VLDLC: Very low density lipoprotein cholesterol; HDL-C/TC: High density lipoprotein cholesterol-total cholesterol ratio; TG/HDL-C: Triglyceride-high density lipoprotein cholesterol ratio; CETP: Cholesterol ester transport protein.

## Competing interests

The authors declare that they have no competing interests.

## Authors’ contributions

FKNA designed the study and participated in drafting manuscript and result analysis. MA-F performed the sample collection, processed the data, as well as conducted statistical analysis and drafted the manuscript. JO-Y, FOM and LO participated in the design of the study and helped in analyzing data and in drafting the manuscript. All authors have read and approved the final manuscript.
